# The Impact of Consequences Awareness of Public Environment on Medicine Return Behavior: A Moderated Chain Mediation Model

**DOI:** 10.3390/ijerph18189756

**Published:** 2021-09-16

**Authors:** Jun Lv, Xuan Liu, Sivhuang Lay

**Affiliations:** 1 Faculty of Economics and Management, East China Normal University, Shanghai 200241, China; sherryliu0730@126.com; 2 Logistics Management, Delivery Hero Co., LTD, Phnom Penh 12309, Cambodia; sivhuang@yahoo.com

**Keywords:** consequences awareness of public environment, personal health awareness, unused or expired medicines, return behavior, chain mediation model

## Abstract

With global aging trends and prosperity in the medicine market, the number of unused or expired household unused or expired medicines is increasing. Medicines which are discarded improperly result in serious pollution. From the perspective of behavioral science, the main contribution of this paper is the construction of a chain mediation model to analyze the influence mechanism between consequences awareness of the public environment and proper return behavior of unused or expired medicines. The model explores the moderating effect of personal health awareness with through observation of to the mediating effect of personal norms and return intention. Using a sample size of 366 residents from China, the proposed hypotheses are empirically tested. The results show: firstly, the direct effect of residents’ consequences awareness of public environmental awareness on the proper medicine return behavior is not significant; secondly, return intention plays a mediating role in the positive effect of consequences awareness of the public environment on proper return behavior; thirdly, personal norms and return intention play a chain mediating role in the positive impact of consequences awareness of the public environment on proper return behavior; and lastly, personal health awareness moderates the chain mediation path by strengthening the positive effect of return intention on proper return behavior.

## 1. Introduction

With increasingly aging populations and the prevalence of chronic diseases, social medicine reserves and the number of unused or expired medicines are rapidly increasing. Unused medicine is a medicine that is still before its expiration date, but is no longer taken before its expiration date, has been forgotten in the corner of the family medicine box, and is likely to become an expired medicine; expired medicine is a medicine that exceeds its shelf life and can no longer be taken. After being taken by mistake, it has no therapeutic effect and may endanger the health of patients. There are cases where patients recover faster, before they have used the medicines they purchased; sometimes, they switch to other treatment regimens and stop using the previously prescribed medicines, resulting in leftover, unfinished drugs which are often within the expiry dates [1]. For diseases or symptoms that can be self-diagnosed, patients independently choose and use medicines for the treatment. Self-medication will lead to unreasonable use and unreasonable storage of medicines [2]. The gap between the use period and the rejection period of medicines leads to the storage of a large number of medicines, bringing about the problems of unused or expired medicines. Categorized as hazardous waste in the household waste return category, unused or expired medicines can pose a serious threat to public environments and personal health if the waste is not properly recycled and destroyed in time [3]. Proper medicine return refers to returning unused or expired medicines stored at home to regular places, such as pharmacies, hospitals, etc., which residents rarely do at present [4]. Improper medicine return refers to directly discarding medicines, such as flushing medicines down the sink or toilet, throwing them in the kitchen bin, selling them, etc., which is the most common medicine return behavior of residents [3,4,5]. Proper medicine return and reuse can minimize hazards to the environment and reduce the overall cost of medicines to our society [6]. Unused or expired medicines can easily decompose, evaporate, emit harmful gases, and grow bacteria. The storage of such medicines is likely to cause indoor pollution and affect personal health. Improper discarding of unused or expired medicines can also cause pollution to the public environment. Many medicines are toxic, and they very easily decompose and release toxins and chemical substances which seriously pollute soil, water systems, and other ecosystems [7,8]. In the United States alone, at least $5 billion worth of unused medicines are thrown away every year [9], which not only wastes a lot of resources, but also brings serious harm to the environment. In addition, a large number of unused or expired medicines are purchased by illegal medicine dealers and sold at low prices in poorer areas, which brings irreparable harm to our human society.

**Figure 1:**
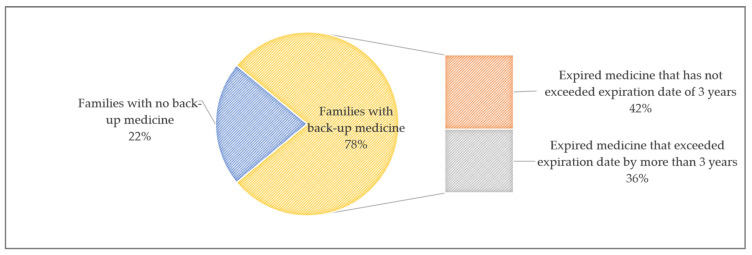
The proportion of expired medicines in Chinese households. Source: The White Paper on the Recycling of Expired Medicines in Chinese Families (2004–2014).

At present, many households have a large stockpile of unused or expired medicines. According to The White Paper on the Recycling of Expired Medicines in Chinese Families (2004–2014), 78.6% of families in China alone have the habit of storing reserve medicines, but about 40% of the medicines are expired, and 30% to 40% of them have exceeded their expiry date by more than 3 years. On average, each family has about 215 expired medicines. However, more than 70% of families did not pay attention to the storage conditions of medicines while about 52.4% of families had small medicine boxes, as shown in Figure 1. The average number of expired medicines in China every year is staggering, exceeding 15,000 tons, and the number is still growing. However, more than 80% of households do not regularly clean up expired medicines, and few people know how to properly dispose of those medicines.

Pharmaceutical products have strict requirements for the supervision and control of various aspects of storage and transportation, such as temperature, return packaging, and transportation time [10]. For many medicines, a temperature deviation of as little as 2 °C can render them stale and worthless. For example, the Live Attenuated Cholera Vaccine has a shelf life of one year when it is stored at temperatures between 2 and 8 °C, but drops to seven days when it is stored at indoor temperatures. Many pharmaceutical companies use sensors to monitor temperatures during shipment and install alarms to alert staff when there are large temperature deviations. A medicine return system differs from other industries due to its complexity and asset specificity. Medicine return is the source and forefront of the medicine return system. Without active participation of the residents in it, the medicine return system cannot be started and operated effectively [11], and, in turn, wholesalers and manufacturers can suffer losses of up to $14 million each year [12]. This points to the importance of residents’ behavior in the reverse supply chain. The insufficient quantity of returned medicines, inappropriately returned medicines, or the failure of residents to properly classify returned medicines are main factors that greatly increase the cost of the system. Why do residents fail to recycle medicines or return medicines adequately? Why are environmentally aware residents still not opting for proper return? Why do residents who have the intention of medicine return not carry out proper medicine return? What are the key factors that affect residents’ proper medicine return behavior? How can we motivate and promote proper medicine return behavior? All these questions deserve further study.

Much of the existing literature focuses on the research of medicine return systems. Kongar found that most pharmaceutical companies contracted medicine return to third-party logistics companies, and information tracking systems can be promoted in the medicine return system. For example, Radio Frequency Identification (RFID) tags can be attached to medicine packages. These are used to identify the authenticity of medicines and extract medicine information to improve the monitoring ability of pharmaceutical enterprises [13]. Khan believed that time is the key to medicine return. The research points out that medicine should be recycled immediately when the medicine still has 10% of its expiration date left. At this time, the value of the medicine is still fully retained and the enterprise can fully tap into the remaining resources. If this time point is missed in medicine return, the medicine will lose all residual value and can only be destroyed [14]. Alshemari found that only when every stakeholder in the medicine return system collaborates effectively can the whole system operate sufficiently. That includes pharmaceutical companies, distributors, retailers, pharmacies, logistics carriers, residents, and government departments [5]. According to the study of Bravo, pharmaceutical enterprises can cooperate with distributors and retailers to recover medicines at regular replenishment so as to reduce the round-trip transportation cost of medicines and optimize the medicine return system [15]. Campos proposed that management, information technology, and infrastructure are key in the medicine return process [16]. Some studies have explored the social responsibility of pharmaceutical enterprises in the closed-loop operation of medicine return and manufacturing [17], the economic benefits of medicine return and reuse to the pharmaceutical supply chain [18,19], and the planning of logistics for medicine return from hospitals [20]. However, there are few studies on residents’ proper return behavior.

Most of the research above was conducted from the perspective of pharmaceutical enterprises and return systems, while few studies were conducted from the perspective of residents and proper residential medicine return behavior. This paper mainly focuses on the return stage of the medicines, which is the start, forefront, and most important part of the whole return system. From the perspective of behavioral science, the main contribution of this paper is to construct a chain mediation model to analyze the influence mechanism between consequences awareness of the public environment and proper return behavior.

Based on the Normative Activation Theory and the Theory of Planned Behavior, residents’ awareness of medicine return is divided into consequences awareness of the public environment outside of the home and personal health awareness inside the home. The mediation model of consequences awareness of the public environment and proper return behavior is constructed, with the mediating role of personal norms and return intention. The moderating role of personal health awareness in this model is also discussed. Based on the theoretical model, this paper empirically tests the proposed hypotheses: (i) the impact of consequences awareness of the public environment on personal norms, return intention, and proper return behavior; (ii) the mediating effect of personal norms and return intention in the impact of consequences awareness of the public environment on proper return behavior; and (iii) the moderating effect of personal health awareness in the chain mediation path. Data is collected through a questionnaire survey, and hypothesis testing is conducted by empirical analysis. With the support of theory and data, the influence mechanism between consequences awareness of the public environment and proper return behavior is deeply analyzed so as to put forward countermeasures and suggestions for promoting residents’ proper medicine return behavior.

## 2. Hypotheses and Conceptual Model

### 2.1. Consequences Awareness of the Public Environment and Proper Return Behavior

Consequences awareness is the tendency to become aware of the consequences of one’s behavior for others in the Normative Activation Theory [21]. Consequences awareness of the public environment refers to residents’ perception of the consequences that medicine return has for the welfare of the public environment. It means residents can realize the consequences of improper medicine return to the public environment outside the home, such as water, air, and soil pollution. Proper return can not only reduce environmental pollution, but can also provide residual value of medicines and save resources. Public environmental resources are typical “quasi-public goods”, which can be shared by the whole society, and are non-exclusive and competitive to a certain extent. The non-exclusivity of environmental resources means that when some residents consume environmental resources, they cannot exclude other residents from consuming resources together, or the cost of exclusion is very high. The competitive nature of environmental resources means that excessive consumption or excessive damage of environmental resources by some residents will affect the utility of other residents’ consumption of these resources to a certain extent [22]. Medicines discarded improperly impose significant negative externalities to the environment because the digestive and circulatory processes of toxic and harmful substances from medicines will bring adverse consequences to environmental resources, which cannot be priced and traded through the market mechanism.

At present, most residents do not have sufficient perception of the harmful consequences of public environmental pollution caused by improperly discarding medicines [23]. They believe that when cleaning up unused medicines or disposing of expired medicines, directly throwing them away together with other garbage is the most effective method, which will not cause serious consequences, such as pollution, to the environment. Some residents mistakenly believe that by selling unused or expired medicines to medicine dealers, they can not only gain economic benefits, but also transfer medicines to people in need. Yet, they are completely unaware of the serious consequences caused by this behavior [24]. Some residents do not even know that there are regular channels and departments for medicine return. As such, residents are misled as to the proper disposal of unused or expired medicines. Residents with a high consequences awareness of the public environment are more aware of the possible harms to the environment and societal welfare caused by their improper medicine return behavior. Therefore, consequences awareness of the public environment is an important factor affecting medicine return behavior. The stronger the residents’ consequences awareness of the public environment, the more willing residents are to carry out proper medicine return behavior. Therefore, the following hypothesis is proposed.

**Hypothesis** **1** **(H1).** *Consequences awareness of the public environment has a positive impact on proper return behavior.*

### 2.2. The Mediating Effect of Personal Norms

Personal norms refer to the responsibility and obligation perceived by an individual when taking a certain behavior, as well as the reflection of an individual’s expectation of a certain behavior and self-value [21]. The theory of Normative Activation Model proposed by Schwartz is mostly used to investigate individual environmental behavior and prosocial behavior. Normative Activation Theory starts from an individual’s cognition of predictable adverse consequences. Such awareness of consequences will trigger personal behavioral norms, which will determine whether an individual should take a specific action to prevent harmful consequences [25].

In this study, the logical thinking of Normative Activation Model is followed, and consequences awareness of the public environment is taken as consequence awareness. Residents are aware that the direct disposal of medicines is an improper return which will cause serious pollution to the external public environment and may threaten the welfare of the whole society. The stronger the awareness of protecting the public environment that residents have, the more they believe that they have responsibilities and obligations in protecting the environment, and they will be prouder of undertaking proper medicine return behavior. This consequences awareness of the public environment can activate residents’ sense of responsibility and obligation for proper medicine return, thus forming residents’ internal code of conduct and values and promoting residents’ behavior of proper medicine return. When residents do not feel that it is their responsibility and obligation, they will not care about the pollution consequences brought to the environment by improperly discarding medicines and will not send unused or expired medicines to regular channels for return. Onwezen’s research found that the awareness of consequences is a key factor that influences individual behavioral norms and indirectly influences behavior [26]. In a study of public energy saving behaviors, Ellen found that the consequences awareness of hazards caused by not saving energy can positively affect the public’s personal norms for energy saving, thus motivating public behaviors of saving energy [27]. In a study on public participation in environmental protection, Han found that the awareness of consequences can activate the public’s personal norms and thus enhance the level of public participation in environmental protection [28]. Hence, the following hypothesis is proposed.

**Hypothesis** **2** **(H2).** *Personal norms play a mediating role between consequences awareness of the public environment and proper medicine return behavior.*

### 2.3. The Mediating Effect of Return Intention

Improper disposal of medicines has a strong negative externality on environment. Because of the non-exclusive and competitive nature of environmental resources, environmental resources available to other residents will be damaged as a consequence of improper medicine disposal while nobody pays any compensation cost. The cost of government’s supervision is high but the effect is very small [22]. Residents with a higher consequences awareness of the public environment can more easily recognize the “quasi-public goods” characteristics of public environmental resources, and will be more able to understand and realize that improper discarding of medicines will damage the public environment. As a result, they are more inclined to return unused or expired medicines properly. Therefore, the higher the consequences awareness of the public environment of residents, the stronger the residents’ proper return willingness will be.

Behavioral intention cannot be equated with behavior. Classical Theory of Planned Behavior proposes that an individual’s behavior is determined by the individual’s intention to carry out or realize a specific behavior [29]. Behavioral intention of medicine return refers to the willingness of residents to return medicines properly. Relative to directly throwing the unused or expired medicines in the trash and sewer, the regular collection points, such as pharmacies and hospitals, usually have a certain distance from residents’ home. Accordingly, people need to take greater efforts or overcome difficulties to send medicines to regular collection points [1]. When residents have a strong intention of return, they are more willing to make efforts to carry out proper medicine return. Therefore, the higher residents’ intention of medicine return, the more they will adopt proper medicine return behavior. Hence, the following hypothesis is proposed.

**Hypothesis** **3** **(H3).** *Return intention plays a mediating role between consequences awareness of the public environment and proper return behavior.*

### 2.4. Chain Mediating Effect of Personal Norms and Return Intention

The Normative Activation Model proposes that consequence awareness activates personal norms and influences behavioral intentions and behaviors. Based on the Normative Activation Theory, this study distinguishes behavioral intention from behavior. This study considers that behavioral intention and behavior are different variables, and behavioral intention positively affects behavior. Zhang studied public transport use behavior of Shanghai residents in 2014 and found that personal norms were an important factor affecting residents’ public transport using intention, and further influenced public transport using behavior [30]. Park’s research in 2014 believed that personal norms influenced the public’s environmentally responsible behavior through behavioral attitudes and intentions [31]. Therefore, based on the Normative Activation Model and the Theory of Planned Behavior, when residents have a strong public awareness of environmental protection and pollution reduction, the consequences awareness of the public environment will activate residents’ personal norms, such as responsibility, obligation, and values, to reduce the negative externalities to the environment. Stronger personal norms will further stimulate a stronger desire for proper medicine return among residents. Under the support of stronger intentions to return medicines properly, residents are more likely to adopt the behavior of proper medicines return. That is to say, consequences awareness of the public environment (consequence awareness) will activate residents’ behavioral norms (personal norms) and then activate residents’ intention to return medicines properly (behavioral intention), and finally affect residents’ proper return of medicines (behavior). Hence, the following hypothesis is proposed.

**Hypothesis** **4** **(H4).** *Personal norms and return intention have a chain mediating effect on the relationship between consequences awareness of the public environment and proper return behavior.*

### 2.5. The Moderating Effect of Personal Health Awareness

Consequences awareness of the public environment focuses on the external environment of the family, while personal health awareness focuses more on the internal environment of the family. Personal health awareness pays more attention to the impact of unused or expired medicines on the health of oneself or family members. Most medicines easily cause indoor environmental pollution after being left unused for a long time or they expire, and even cause harm to the human respiratory tract. At a certain temperature, medicines with excipients, such as granules and honey pills, will develop mildew and grow bacteria, which is difficult to identify with our eyes only [32]. Taking expired medicines by mistake may lead to antibiotic resistance, treatment failure, carcinogenicity, and other problems, which can endanger individual health to varying degrees [33]. Personal health awareness helps residents objectively understand the harm of unused or expired medicines so as to promote self-control and regulation of intentions and behaviors. Therefore, this study believes that there is an interaction between personal health awareness and medicine return intention, which will have a profound impact on residents’ consequence awareness, personal norms, and behaviors.

First of all, personal health awareness will strengthen the positive effect of return intention on proper return behavior. The home environment is the main living place of residents, and the family members are the main concern of residents. According to the survey, 96.7% of respondents believe it is a family responsibility to prevent family members from getting into contact with harmful or expired medicines [34]. Concern for the home environment and the health of the family promotes a better understanding and acceptance by the population of the possible risks to personal health caused by unused or expired medicines. When it is found that there are too many unused or expired medicines stored at home, residents may postpone or even give up the proper return behavior due to the lack of convenient regular return channels despite their intention to carry out proper return behavior of these unused or expired medicines. At this point, residents with high personal health awareness realize that unused or expired medicines will bring serious health hazards to themselves and their families. Therefore, they will make more efforts to overcome the difficulties and turn the behavioral intention of proper return into real return behavior.

Secondly, personal health awareness will strengthen the positive impact of return intention on proper return behavior, and play a moderating role on the chain mediation effect of the theoretical model. Specifically, when personal health awareness is strong, residents’ concern for their home environment and family health will guide residents to continuously strengthen their self-control and adjustment ability, which may encourage residents to compare different environmental consequences and strengthen the formation of their personal code of conduct and values. Thus, the return intention can be promoted to transform into proper return behavior. Therefore, the stronger the personal health awareness is, the stronger the positive impact of return intention on proper return behavior can be, demonstrating the chain mediating role of personal norms and return intention between consequences awareness of the public environment and return behavior. Hence, the following hypotheses are proposed.

**Hypothesis** **5** **(H5).** *Personal health awareness moderates the positive impact of return intention on proper return behavior. The stronger personal health awareness is, the greater the impact of return intention on proper return behavior is.*

**Hypothesis** **6** **(H6).** *Through strengthening the positive effect of return intention on proper return behavior, personal health awareness moderates the chain mediating effect of personal norms and return intention on the relationship between consequences awareness of the public environment and proper return behavior.*

**Figure 2:**
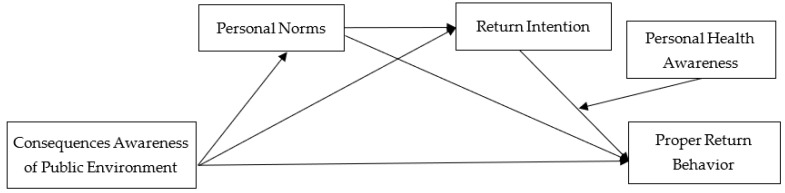
Theoretical research model.

In conclusion, this study builds a chain mediation path based on Normative Activation Theory combined with the Theory of Planned Behavior. The model takes consequences awareness of the public environment as the independent variable, uses proper return behavior as the dependent variable of behavior, and takes personal norms and return intention as the norms and the behavioral intention. The path is shown as “Consequences awareness of the public environment–Personal norms–Return intention–Proper return behavior”, in which personal health awareness’ moderating effect in this chain mediation is discussed. The conceptual model of this study is shown in Figure 2.

## 3. Methodology

### 3.1. Questionnaire Design

In order to ensure the reliability and validity of the questionnaire, all the scales required in this study are well-established scales published in domestic and foreign core journals and cited many times. All English scales adopt standard translation–retranslation procedures, and a research group is set up to revise the questionnaires to avoid semantic fuzziness or ambiguity as much as possible. All the variables involved in this paper are measured by a Likert 5-point scale in which 1 represents “strongly disagree”, 2 represents “disagree”, 3 represents “general”, 4 represents “agree”, and 5 represents “strongly agree”.

The measurement of consequences awareness of the public environment was designed by referring to the research of Yan, Munerah, Kautish and Zhang [35,36,37,38], including 3 items. For example, “Discarding medicines improperly will cause serious pollution to the environment”, “Discarding medicines will cause much more pollution to the environment than I had realized”, etc. For the measurement of personal norms, refer to the scales in Onwezen and Han [26,39]. There are 4 items in total, for example, “I think it is a social virtue to recycle my unused or expired medicines”, “I should do something to recycle my unused or expired medicines”, etc. The personal health awareness was measured by referring to the Outcome Awareness Scale in the study of Zhang and Shi [40,41], with a total of 4 items. For example, “I think there is a risk of accidental ingestion of expired medicines if they are not handled in time”, “I think that the direct disposal of medicines in rubbish bins or sewers may affect my personal health”, etc. The measurement of return intention was designed by referring to the research of Bezzina and Pei [42,43], consisting of 4 items. For example, “I am willing to collect unused or expired medicines stored at home and take them for return”, “I am willing to encourage my relatives and friends to participate in medicine return activities”, etc. The measurement of proper return behavior refers to the scale developed by Onwezen and Park [26,31], consisting of three items. For example, “I have the experience of taking unused or expired medicines for return”, “I have the experience of publicizing or being informed about the knowledge of medicine return”, etc. All the scales and items are shown in Table A1 of Appendix A.

The original questionnaire of this study consists of two parts. The first part is the scale of consequences awareness of the public environment, personal norms, personal health awareness, return intention, and proper return behavior, with a total of 17 items. The second part is demographic information, including gender, age, education, career, and monthly income.

After the initial design and improvement of the scale, 99 valid questionnaires were collected for pre-test in this study to analyze the reliability and validity. The results show that the internal consistency coefficient of the total scale is 0.879, and the internal consistency coefficient of each subscale is 0.770, 0.717, 0.842, 0.899, 0.787, respectively, which are all greater than 0.7. The reliability of each scale is good. The KMO value of the total scale is 0.829, the Sig value of the Bartlett sphericity test is less than 0.001, and the total variance of factor explanation is 75.376%. The result of the validity test is acceptable. In the pre-test stage, reasonable modifications are made to the questionnaire and scale items by referring to the suggestions of some subjects, so as to make it easier for the subjects to understand, and formed the final version of the questionnaire.

### 3.2. Data Collection and Sample

In this research, the questionnaire was officially distributed online and offline, and the respondents were mainly from China. It took eight months from issuing to collecting questionnaires, and a total of 430 questionnaires were finally collected. After excluding invalid questionnaires with continuous choice of the same answer, obvious regularity of answers, and wrong answers to key lie test questions, 366 valid questionnaires were obtained, with a high response rate of 85.12%.

In the sample, male subjects account for 48.28% and female subjects account for 51.72% of respondents. The age of the subjects is mainly between 18 and 40 years old, accounting for 92.12% of respondents. Most of the subjects have a high education level, accounting for 59.18% with a bachelor’s degree, 29.15% with a master’s degree or above, 5.54% with a high school degree or below, and 6.12% with college degree. In terms of monthly income, most of the subjects’ monthly income is 1378 USD or less, accounting for 91.84% of respondents.

## 4. Empirical Results

### 4.1. Reliability and Validity

Firstly, the reliability of the scale is tested by calculating the internal consistency coefficient of the scale. Among them, the internal consistency coefficient of consequences awareness of the public environment is 0.789, the internal consistency coefficient of personal norms is 0.887, the coefficient of personal health awareness is 0.741, return intention’s coefficient is 0.901, and as for proper return behavior, the coefficient is 0.883. The internal consistency coefficient of the total scale is 0.890, greater than 0.7, indicating that the scale this paper used has good reliability. Secondly, factor analysis is conducted on the total scale, and the KMO value is 0.895. The results of the Bartlett sphericity test are all significant at the level of *p* < 0.001, indicating a good scale validity. The cumulative variance contribution rate is 75.839%, indicating that each variable could effectively reflect the original data content after factor analysis.

### 4.2. Common Method Deviation Test

Since the self-assessment approach may lead to the common method bias among the five constructs, this paper first tested the common method bias problem. Firstly, Harman’s single-factor method was used for testing. After an unrotated exploratory factor analysis for all items of the research variables, the total amount of explanation for variation of the factors was 75.839%, among which the amount of explanation for variation of the first principal component was 36.605%, which is not more than 50% of the maximum and less than half of the total amount of explanation for variation. Therefore, there is no serious common method bias problem where a single factor explains most of the variance of all variables.

In order to test the degree of differentiation among key variables, this paper uses AMOS 22.0 to conduct confirmatory factor analysis on key variables, and the results are shown in Table 1. According to the analysis results in Table 1, the fitting indexes of the five-factor model were significantly better than those of other comparative models ($\chi^{2}/df$ = 2.740, CFI = 0.949, TLI = 0.936, IFI = 0.949, RMSEA = 0.069), which indicated that the five variables studied in this paper had good discriminative validity. Each variable represents a different construct. Therefore, the deviation of the common method in this study is at an acceptable level and will not have a serious impact on the results of the study.

### 4.3. Correlation Analysis

The mean value, standard deviation, and correlation coefficient of the variables involved in this paper are shown in Table 2. According to the table below, it can be seen that:

- Consequences awareness of the public environment had a positive correlation with personal norms (r = 0.530, *p* < 0.01), personal health awareness (r = 0.563, *p* < 0.01), return intention (r = 0.553, *p* < 0.01), and proper return behavior (r = 0.248, *p* < 0.01).
- There was a significant positive correlation between personal norms and personal health awareness (r = 0.507, *p* < 0.01), and personal norms had a significantly positive correlation with return intention (r = 0.638, *p* < 0.01), and was positively correlated with proper return behavior (r = 0.242, *p* < 0.01).
- Personal health awareness was significantly positively correlated with return intention (r = 0.385, *p* < 0.01) and normal return behavior (r = 0.242, *p* < 0.01).
- There was a significant positive correlation between return intention and proper return behavior (r = 0.284, *p* < 0.01).

The results of correlation analysis are in accordance with our expectations, which preliminarily verifies the research hypothesis proposed in this paper.

### 4.4. Hypothesis Test

#### 4.4.1. Mediation Model Test

**Figure 3:**
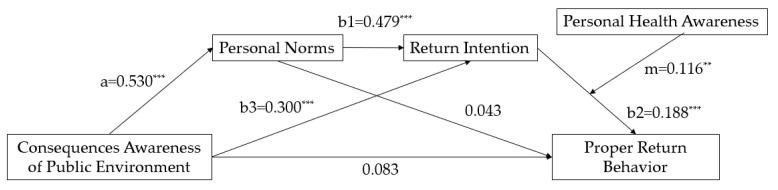
Estimation of moderated mediation model. Note: *** *p* < 0.01, ** *p* < 0.05.

In this paper, Mplus 8.0 was used to test the mediating effect of the model by combining the structural equation model with the Bootstrap method. The operation results of the theoretical model and the Bootstrap test results were shown in Figure 3 and Table 3.

The path coefficient between consequences awareness of the public environment and proper return behavior is not significant (β = 0.083, *p* = 0.173), indicating that the direct impact of consequences awareness of public the environment on proper return behavior is not significant. Hypothesis 1 is not supported. The path coefficient between personal norms and consequences awareness of the public environment (a) is 0.530 (*p* < 0.001), indicating that consequences awareness of the public environment can significantly positively predict personal norms. The path coefficient between personal norms and proper return behavior is not significant (β = 0.043, *p* = 0.481). Therefore, the mediating effect of consequences awareness of the public environment on return behavior through personal norms is not significant. Therefore, hypothesis 2 is not supported.

The path coefficient of consequences awareness of the public environment on return intention (b3) is 0.300(*p* < 0.001), indicating that consequences awareness of the public environment has a significant positive effect on return intention. The path coefficient of return intention to proper return behavior (b2) is 0.188 (*p* < 0.001), indicating that return intention has a significant mediating effect between consequences awareness of the public environment and proper return behavior (β = 0.056, *p* < 0.01), and the 95% confidence interval of Bootstrap = 5000 was [0.023, 0.111], excluding 0. Hypothesis 3 was verified.

At the same time, the path coefficient from personal norms to return intention (b1) is 0.479(*p* < 0.001), indicating that personal norms have a significant positive impact on return intention. Combined with the hypotheses verified above, it can be seen that personal norms and return intention have a significant chain mediating effect between consequences awareness of the public environment and proper return behavior (β = 0.048, *p* < 0.05), and the 95% confidence interval of Bootstrap = 5000 was [0.017, 0.096], excluding 0. Therefore, hypothesis 4 was verified.

#### 4.4.2. Moderating Effect Test

In this paper, with reference to the moderated chain mediation model algorithm proposed by Stride [44], Mplus 8.0 is used to test the moderating effect of the model. The results are shown below.

**Figure 4:**
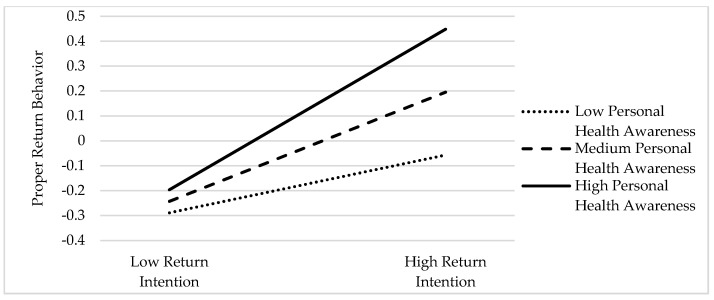
The analysis of moderating effect.

First, the interaction of return intention and personal health awareness had a significant positive influence on proper return behavior (β = 0.116, *p* < 0.01), indicating that personal health awareness significantly moderated the relationship between return intention and proper return behavior. In order to further explain the moderating effect, we conducted a simple slope analysis through Mplus 8.0, and drew the moderating effect analysis diagram as shown in Figure 4. The results showed that when the level of personal health awareness was low, the effect of return intention on proper return behavior was not significant. When individuals have a medium level of personal health awareness, return intention has a weak positive effect on proper return behavior. When individuals have a stronger personal health awareness, return intention has a strong positive effect on proper return behavior. This shows that when individuals have a strong personal health awareness, the positive effect of return intention on proper return behavior will be enhanced. Thus, Hypothesis 5 was verified.

Then, the moderated chain mediating effect was further analyzed under different conditions. Whether the moderated mediating effect was significant through testing the significance of the product of the path coefficient between interaction terms and mediator was judged. The results are as follows: in the chain mediation in which consequences awareness of the public environment influences proper return behavior through personal norms and return intention, the product of path coefficient (a × b1 × m) between interaction term and mediating variable is 0.029 (*p* < 0.05), and the 95% confidence interval of Bootstrap = 5000 was [0.012, 0.060], excluding 0, indicating that the chain mediating effect was regulated by personal health awareness. In Table 4, when personal health awareness is low, the mediating effect of consequences awareness of the public environment on proper return behavior through personal norms and return intention is not significant. When personal health awareness was at a medium level, the indirect effect of consequences awareness of the public environment in the chain mediation model was 0.048 (*p* < 0.05), while the 95% confidence interval is [0.017, 0.096] and does not contain 0, which means that the chain mediated effect is significant. When individuals had a strong health awareness, the chain mediating effect was 0.077 (*p* < 0.01), the 95% confidence interval is [0.036, 0.140] and excludes 0, showing that the chain mediated effect is significant. The results showed that the chain mediating effect of personal norms and return intention on the relationship between consequences awareness of the public environment and proper return behavior was significantly enhanced when residents’ personal health awareness level was higher. Hypothesis 6 is verified. Results of hypothesis testing are presented in Table 5.

## 5. Discussion

### 5.1. The Direct Impact of Consequences Awareness of the Public Environment on Proper Return Behavior

The positive effect of consequences awareness of the public environment on proper return behavior is not significant, that is, higher consequences awareness of the public environment does not necessarily lead to higher proper return behavior. This is consistent with the results of Manocha’s survey in 2020 [33]. Manocha conducted a questionnaire survey on the public’s understanding and handling of unused or expired medicines. Most respondents were aware of the necessity of return unused or expired medicines, but only 6% of them were in accordance with the requirements, choosing more environmentally friendly ways to return medicines to the pharmacy or collection points. More people still throw their medicines away directly, with 73% throwing them in the bin and 20% flushing them in the toilet [34]. The direct effect of consequences awareness of the public environment on proper return behavior is not significant, but it has a significant indirect effect through mediating variables. Therefore, it is not sufficient for residents to improve their consequences awareness of the public environment through public environmental publicity and pro-environmental education only, which may not be effective in promoting proper medicine return behavior. Personal norms, return intention, personal health awareness, and other aspects of guidance, cultivation, and incentive measures or means need to be combined in order to effectively promote the improvement of proper return rates.

### 5.2. The Mediating Effect of Personal Norms and Return Intention

Although the indirect effect of consequences awareness of the public environment on return behavior through personal norms is not significant, the indirect effect of consequences awareness of the public environment on return behavior through return intention is significant. Personal norms had no significant mediating effect on the relationship between consequences awareness of the public environment and return behavior. Residents with strong consequences awareness of the public environment are more likely to form personal norms such as responsibility, obligation, and intrinsic values for environmental protection. Residents with consequences awareness of the public environment are more able to understand the non-exclusivity of environmental resources and the “negative externalities” brought to the environment by the disposal of medicines. Hence, they can form strong personal norms and proper return intentions. However, the direct influence of personal norms on proper medicine return behavior is not significant, and there is still an indirect relationship between personal norms and proper medicine return behavior. Return intention has a significant positive mediating effect between consequences awareness of public environment and return behavior. Residents with a strong awareness of the public environment are more inclined to carry out proper return of medicines. Driven by a strong return intention, residents will make more practical efforts to return medicines to the regular channels.

Personal norms and return intentions have significant chain mediating effects on the relationship between consequences awareness of the public environment and proper return behavior. With stronger consequences awareness of the public environment, residents are more likely to form stronger personal norms. Residents are also more likely to realize that proper medicine return is in line with their expectations and values, which can promote their willingness to perform proper medicine return. The stronger the willingness of residents to perform proper medicine return is, the more likely they are to carry out proper medicine return behavior.

### 5.3. The Moderating Effect of Personal Health Awareness

Personal health awareness significantly moderated the positive effect of return intention on proper return behavior. When residents perceive that the hazards to personal health from unused or expired medicines are low, the positive effect of return intention on proper return behavior is not significant, and residents with return intention may not undertake proper medicine return behavior. When personal health awareness is gradually raised to medium and high levels, the positive effect of return intention on proper return behavior is significant and increasing. The moderating effect of personal health awareness on the chain mediating path of “consequences awareness of the public environment—personal norms—return intention—proper return behavior” was also significant. When residents perceived personal health hazard from unused or expired medicines is low, the correlation between consequences awareness of the public environment and proper return behavior was not significantly mediated by personal norms and return intention. That is to say, for low personal health awareness, the improvement of consequences awareness of the public environment cannot necessarily promote the increase of proper return behavior through personal norms and return intention. When the residents’ personal health awareness increases to a medium or high level, the mediating effect of personal norms and return intention is significant and continuously strengthened. The increase in personal health awareness significantly strengthened the mediating effect of return intention and the chain mediating effect of personal norms and return intention, which prompted more residents to carry out proper return behavior for unused or expired medicines.

### 5.4. Implications for Medicine Return Management

In our research, consequences awareness of the public environment has a significant indirect effect on proper return behavior through the mediating variables of personal norms and return intention. As a result, more emphasis should be placed on residents’ responsibilities and obligations and the construction of values to cultivate consequences awareness of the public environment of medicine return among residents, which can promote the formation of return intention. Extensive publicity and education should be carried out in all cities and regions, aiming to make residents fully realize that proper medicine return behavior is not only the responsibility of the government or enterprises, but also the responsibility and obligation of every citizen in the society. As for environmental resources, which are “quasi-public goods”, if every resident believes that proper medicine return is another’s responsibility, the “tragedy of the commons” will easily occur. Only when residents’ sense of responsibility is improved together can the health and safety of the public environment be guaranteed and social resources be saved. Relevant departments can establish a long-term stable public communication mechanism, comprehensively utilizing public relations, radio, television, network, and other media. These measures can make residents perceive the importance and universality of medicine return activities and understand the regular medicine return channels and methods. As a consequence, residents could be more willing to undertake proper medicine return activities and improve their own proper medicine return behavior by themselves.

Our results have shown that personal health awareness has a significantly moderating role in the chain mediation model, so residents’ awareness of personal health and family environmental health need to be cultivated. Moreover, residents’ correct concept of medicine use and the concept of proper return and disposal of expired medicines need to be established. On the one hand, residents should be guided to develop the habit of purchasing medicines in appropriate amounts. The government should establish consumers’ concepts of green environmental protection through media publicity so that residents will not buy or store too many medical products, nor will they sell medicines no longer needed to illegal vendors for small profits, and voluntarily send them to regular return points. Doctors, pharmacies, and medicine retailers, when prescribing or selling medicines to patients, should popularize the knowledge of proper medicine return and healthy medicine use habits to residents, and regular return sites also should encourage residents to return unused or expired medicines when purchasing medicines. The public influence of licensed pharmacists can be improved so that residents can seek the guidance of licensed pharmacists when purchasing medicines and try to avoid excessive purchasing of medicines. On the other hand, residents should be guided to properly reserve unused medicines and develop the habit of cleaning up expired medicines regularly. Families should always have a professional medicine storage box to classify and place medicines. The medicine box should be placed in a dry place away from light to avoid the failure of medicines due to environmental influences such as temperature and humidity. Each medicine should be stored with its outer packaging to reduce medicine confusion and inability to identify expiration dates. The expired medicines in the box should be cleaned every 3–6 months to avoid being taken by mistake or contaminating other medicines.

Finally, many cities have not set up special return points for medicines and many medicine return activities are short-term and occasional, yet need to be continuous and standardized. Due to the uniqueness and diversity of medicines, pharmaceutical companies would be more aware of the medicines they produce and create more professional and effective return capabilities. Hence, the producer responsibility system of “who produces, who is responsible” can be introduced into medicine return, and the government can clearly define the responsibilities and obligations of pharmaceutical enterprises, wholesale, and retail entities in the downstream channels to optimize the return system. The government can also establish special return channels and set up special medicine return drop boxes in each community, which are dedicated to medicine delivery and cannot be opened at will, so as to improve the convenience of medicine return. Relevant departments of society, environment, and health can also encourage more social forces to participate in the medicine return system. These forces can contribute to establish safe and reliable medicine return channels and platforms for unused or expired medicines, and promote the long-term implementation of the unused or expired medicines return mechanism. Government can clear the return and supervision main body and return channel through laws and regulations, making sure that there are legal bases for medicine returns, and further ensure that the discards and illicit circulation of medicines are reduced, thus stopping improper return and illegal acquisition from different sources to ensure the safety of the social environment. All these can put an end to the harm caused by the outflow of unused or expired medicines to society and form a standardized and guaranteed long-term return mechanism.

### 5.5. Limitations and Future Research

First, future research can expand the survey scope of research objects, cover different countries and regions in the world as far as possible, and explore the influence of variables that reflect national and regional differences on the chain mediating effect and moderating effect of proper medicine return, such as demographic variables, cultural living habits, climate differences, etc., to further improve the predictive and explanatory power of research on residents’ medicine return behavior.

Second, future research could separate the unused medicines and expired medicines, treat them as two types of household reserve medicines for a comparative study, discuss the similarities and differences of residents’ return behavior of unused medicines and expired medicines, and study the model improvement and suggested measures for the two types of medicines.

Lastly, future research could study the intention and behavior of pharmaceutical enterprises participating in medicine return from the perspective of pharmaceutical enterprises, carry out the extended responsibility system of pharmaceutical manufacturers, promote pharmaceutical enterprises to recycle their own medicines, and conduct research on the closed-loop supply chain of medicine industry return and reuse.

## 6. Conclusions

Most of the previous research focuses on the optimization and operation mode of the medicine return system. There is still little research on the formation mechanism, occurrence mechanism, and promotion measures of residents’ proper medicine return behavior from the perspective of behavioral science. The main contribution of this paper is to study the influence mechanism between consequences awareness of the public environment and proper return behavior from the perspective of behavioral science. This study provides a new research perspective for thinking about medicine return and serves as a supplement and enrichment to previous studies.

In this study, residents’ awareness of medicine return was divided into consequences awareness of the public environment outside the home and personal health awareness inside the home. Based on the Normative Activation Theory and the Theory of Planned Behavior, a theoretical model was built to deeply analyze the mechanism of action between environmental awareness and proper return behavior.

The results show that the direct positive effect of consequences awareness of the public environment on proper return behavior is not significant, but the mediating effect of return intention between consequences awareness of the public environment and return behavior is significant. The chain mediation effect of consequences awareness of the public environment on proper return behavior through personal norms and return intention is significant. Personal health awareness significantly moderates the positive effect of return intention on proper return behavior and the chain mediation effect. For the purpose of promoting proper medicine return behavior, it is not enough to improve residents’ consequences awareness of the public environment only, as it needs to jointly improve residents’ personal norms, return intentions, and personal health awareness. Furthermore, the study provides practical suggestions for cultivating residents’ consequences awareness of the public environment, personal norms, return intentions, and personal health awareness, and for optimizing the medicine return system.

## Figures and Tables

**Table 1:** Confirmatory factor analysis results.

Model	$\chi^{2}/df$	CFI	TLI	IFI	RMSEA
Five-Factor Model: CAPE, PN, PHA, RI, PRB	2.740	0.949	0.936	0.949	0.069
Four-Factor Model: CAPE, PN + PHA, RI, PRB	4.189	0.903	0.883	0.903	0.093
Three-Factor Model: CAPE + PN + PHA, RI, PRB	5.246	0.867	0.844	0.868	0.108
Two-Factor Model: CAPE + PN + PHA + RI, PRB	7.769	0.785	0.752	0.786	0.136
One-Factor Model: CAPE + PN + PHA + RI + PRB	12.975	0.616	0.561	0.618	0.181

Note: CAPE, Consequences awareness of the public environment; PN, Personal Norms; PHA, Personal Health Awareness; RI, Return Intention; PRB, Proper Return Behavior.

**Table 2:** Descriptive statistics and correlations among variables.

	Mean	SD	1	2	3	4	5
PEA	4.380	0.662	1				
PN	4.288	0.687	0.530 **	1			
PHA	4.313	0.717	0.563 **	0.507 **	1		
RI	4.200	0.746	0.553 **	0.638 **	0.385 **	1	
PRB	2.627	1.331	0.248 **	0.242 **	0.242 **	0.284 **	1

Note: N = 366; ** *p* < 0.05; CAPE, Consequences awareness of the public environment; PN, Personal Norms; PHA, Personal Health Awareness; RI, Return Intention; PRB, Proper Return Behavior.

**Table 3:** Mediating effect and 95% confidence interval estimated by Bootstrap method.

Path		Indirect Effect Estimation	CI at 95% Level	
Total indirect effect		0.127	0.060	0.203
Indirect effect	CAPE-PN-PRB	0.023	−0.042	0.084
	CAPE-RI-PRB	0.056	0.023	0.111
	CAPE-PN-RI-PRB	0.048	0.017	0.096

Note: CI, Confidence Interval; CAPE, Consequences awareness of the public environment; PN, Personal Norms; PHA, Personal Health Awareness; RI, Return Intention; PRB, Proper Return Behavior.

**Table 4:** The analysis of moderated chain mediation effect.

Moderator	Path: CAPE-PN-RI-PRB		
	Indirect Effect	CI at 95% Level	
		Lower Limit	Upper LIMIT
Low PHA	0.018	−0.015	0.063
Medium PHA	0.048	0.017	0.096
High PHA	0.077	0.036	0.140

Note: CI, Confidence Interval; CAPE, Consequences awareness of the public environment; PN, Personal Norms; PHA, Personal Health Awareness; RI, Return Intention; PRB, Proper Return Behavior.

**Table 5:** Results of hypothesis testing.

Hypotheses	Results
**Hypothesis 1 (H1).** *Consequences awareness of the public environment has a positive impact on proper return behavior.*	Not Supported
**Hypothesis 2 (H2).** *Personal norms plays a mediating role between consequences awareness of the public environment and proper medicine return behavior.*	Not Supported
**Hypothesis 3 (H3).** *Return intention plays a mediating role between consequences awareness of the public environment and proper return behavior.*	Supported
**Hypothesis 4 (H4).** *Personal norms and return intention have a chain mediating effect on the relationship between consequences awareness of the public environment and proper return behavior.*	Supported
**Hypothesis 5 (H5).** *Personal health awareness moderates the positive impact of return intention on proper return behavior.*	Supported
**Hypothesis 6 (H6).** *Personal health awareness moderates the chain mediating effect of personal norms and return intention on the relationship between consequences awareness of the public environment and proper return behavior.*	Supported

## Data Availability

Data are available on reasonable request.

## Appendix A

**Table A1:** Survey on Medicines Recycling Behavior.

Scales	Items
Consequences Awareness of the Public Environment	Discarding medicines improperly will cause serious pollution to the environment. 1 (strongly disagree) to 5 (strongly agree)
	Discarding medicines will cause much more pollution to the environment than I had realized. 1 (strongly disagree) to 5 (strongly agree)
	Recycling unused or expired medicines from home can reduce environmental pollution. 1 (strongly disagree) to 5 (strongly agree)
Personal Norms	I think it is a social virtue to recycle my unused or expired medicines. 1 (strongly disagree) to 5 (strongly agree)
	I should do something to recycle my unused or expired medicines. 1 (strongly disagree) to 5 (strongly agree)
	I think that recycling unused or expired medicines is my contribution to the environment. 1 (strongly disagree) to 5 (strongly agree)
	I feel that I have done a great service to society by recycling the medicines that are no longer used. 1 (strongly disagree) to 5 (strongly agree)
Personal Health Awareness	I think there is a risk of accidental ingestion of expired medicines if they are not handled in time. 1 (strongly disagree) to 5 (strongly agree)
	I think that the direct disposal of medicines in rubbish bins or sewers may affect my personal health. 1 (strongly disagree) to 5 (strongly agree)
	I think taking medicines that have been idle for a long time or have soon expired has bad effects to my health. 1 (strongly disagree) to 5 (strongly agree)
Return Intention	I am willing to collect unused or expired medicines stored at home and take them for return. 1 (strongly disagree) to 5 (strongly agree)
	I am willing to encourage my relatives and friends to participate in medicine return activities. 1 (strongly disagree) to 5 (strongly agree)
	I am willing to participate in proper medicines recycling activities. 1 (strongly disagree) to 5 (strongly agree)
	I am willing to publicize and promote proper medicines recycling activities. 1 (strongly disagree) to 5 (strongly agree)
Proper Return Behavior	I have the experience of taking unused or expired medicines for return. 1 (strongly disagree) to 5 (strongly agree)
	I have the experience of publicizing or being informed about the knowledge of medicine return. 1 (strongly disagree) to 5 (strongly agree)
	I used to recycle unused or expired medicines properly and regularly. 1 (strongly disagree) to 5 (strongly agree)
